# Long-Term Exposure to Microcystin-LR Induces Gastric Toxicity by Activating the Mitogen-Activated Protein Kinase Signaling Pathway

**DOI:** 10.3390/toxins15090574

**Published:** 2023-09-18

**Authors:** Ying Liu, Yafang Li, Qinmei Tan, Yilin Lv, Yan Tang, Yue Yang, Xueqiong Yao, Fei Yang

**Affiliations:** 1 Hunan Province Key Laboratory of Typical Environmental Pollution and Health Hazards, School of Public Health, Hengyang Medical School, University of South China, Hengyang 421009, China; liuying@usc.edu.cn (Y.L.); lyf2022@stu.usc.edu.cn (Y.L.); 20222014111352@stu.usc.edu.cn (Q.T.); lylnhdx@126.com (Y.L.); jiayi1530@163.com (Y.T.); 2 The First Affiliated Hospital, Hengyang Medical School, University of South China, Hengyang 421009, China; yangy930806@126.com; 3 Laboratory of Ecological Environment and Critical Human Diseases Prevention of Hunan Province, School of Basic Medical Sciences, Hengyang Medical School, University of South China, Hengyang 421009, China

**Keywords:** MC-LR, gastric toxicity, inflammation, MAPK signaling pathway, mitophagy

## Abstract

Previous studies have primarily concentrated on the hepatotoxicity of MC-LR, whereas its gastric toxicity effects and mechanisms of long-term exposure under low dosage remain unknown. Herein, the gastric tissue from C57BL/6 mice fed with drinking water contaminated by low-dose MC-LR (including 1, 60, and 120 μg/L) was investigated. The results obtained showed that exposure to different concentrations of MC-LR resulted in significant shedding and necrosis of gastric epithelial cells in mice, and a down-regulation of tight junction markers, including ZO-1, Claudin1, and Occludin in the stomach, which might lead to increased permeability of the gastric mucosa. Moreover, the protein expression levels of p-RAF/RAF, p-ERK1/2/ERK1/2, Pink1, Parkin, and LC3-II/LC-3-I were increased in the gastric tissue of mice exposed to 120 μg/L of MC-LR, while the protein expression level of P62 was significantly decreased. Furthermore, we found that pro-inflammatory factors, including IL-6 and TNF-ɑ, were dramatically increased, while the anti-inflammatory factor IL-10 was significantly decreased in the gastric tissue of MC-LR-exposed mice. The activation of the MAPK signaling pathway and mitophagy might contribute to the development of gastric damage by promoting inflammation. We first reported that long-term exposure to MC-LR induced gastric toxicity by activating the MAPK signaling pathway, providing a new insight into the gastric toxic mechanisms caused by MC-LR.

## 1. Introduction

Recently, cyanobacterial bloom outbreaks have become a major public health concern worldwide [1,2]. These blooms release microcystins (MCs), a cyclic heptapeptide hepatotoxin, which can adversely affect the balance of aquatic ecosystems and people’s health [3]. Exposure to water contaminated with MCs has caused the deaths of 60 patients in Brazil [4]. Moreover, it has been reported that the concentration of MCs enriched in vegetables can be as high as 382 μg/kg, which can also be enriched in large quantities in aquatic products such as crops, fruits, and fish [5,6,7]. Compared with 278 MC isomers, microcystin-LR (MC-LR) is a widely distributed MC, which exhibits the highest toxicity via various exposure routines such as ingesting contaminated food or water and inhaling aerosols containing MC-LR [8,9,10,11]. Exposure to MC-LR causes damage to various target organs in the body, including the liver [12,13], gonads [14], kidneys [15], heart [16], brain [17], intestinal tract [18,19], and other tissues. Consequently, the International Cancer Research Center (IARC) classifies MC-LR as a Group 2B carcinogen [20]. In addition, the drinking water limitation of MC-LR is 1 μg/L, established by the World Health Organization (WHO), based on data from mice (gavaged 40 μg/kg BM/d of MC-LR for 13 weeks) [21], which is around 270 μg/L in mice [22]. However, our previous work confirmed that 60 μg/L of MC-LR can lead to the mutual regulation between tight junctions and inflammatory responses in the intestinal tract [19], suggesting that it is imperative to re-visit the uptake of MC-LR and its toxicity in vivo.

While the intestinal mucosal barrier, including mucosal epithelial cells and lamina propria, is regarded as the main tissue for absorbing MC-LR, partial absorption in the stomach should be underlined [23]. Studies have shown that MC-LR can be translocated into a variety of cells through the organic anion-transporting polypeptide (OATP), which is expressed in gastric tissue, including OATP2B1, OATP2A1, and OATP1B2 [24,25], indicating that gastric tissue cells could absorb the MC-LR. Meanwhile, MCs have been detected in the stomach after intravenous administration of MC-LR to rats and have caused damage to the epithelial surface in medaka fish embryos and epithelial exfoliation and necrosis of epithelial cells in the rat stomach when exposed orally to a mixture of CYN and MC-LR (75 + 750 µg/kg) [26,27,28]. Epidemiological studies have also linked MC-LR exposure with gastrointestinal symptoms and increased gastric cancer risk [29,30]. Hence, MC-LR accumulation in gastric tissue cells would greatly threaten the gastric tissue. Despite these findings, research conducted to investigate the underlying mechanism of gastric toxicity from MC-LR ingestion is limited.

Previous studies have identified serine/threonine PPs, such as PP1 and PP2A, as targets of MC-LR [31,32,33]. PP2A is primarily responsible for dephosphorylating phosphoserine/threonine proteins, thus regulating various signaling pathways’ transduction [34]. It has been shown that PP2A was linked to cell-tight junctions, which could disrupt certain epithelial monolayers by activating extracellular signal-regulated kinase (ERK) [35]. The most important components of tight junctions (TJs) are ZO-1, Claudin-1, and Occludin [36]. We previously demonstrated that chronic MC-LR exposure led to the disruption of the colorectal epithelial barrier in mice and found that there might be mutual regulation between tight junctions and inflammatory responses [19]. However, as a potent and specific PP2A inhibitor, the mechanism of PP2A in MC-LR-induced gastric toxicity by disrupting gastric epithelial cell tight junctions is still unclear [31]. Studies showed that MC-LR activated the p38/MAPK signaling pathway by inhibiting the activity of PP2A [37,38,39,40]. PP1 and PP2A inhibition irreversibly disrupts the protein phosphorylation/dephosphorylation homeostasis, which further activates ERK1/2 of the mitogen-activated protein kinase (MAPK) signaling pathway [41], leading to damage to the digestive system. However, MC-LR-induced gastric injury through the MAPK signaling pathway remains unclear. Moreover, studies demonstrated that the MAPK signaling pathway was related to mitophagy, which is an essential mechanism for maintaining mitochondrial homeostasis [42,43,44].

To date, there is limited evidence for gastric toxicity from long-term environmental exposure to MC-LR. Previous studies focused on the hepatotoxicity and enterotoxicity of chronic MC-LR exposure [19,45,46]. Herein, we established a male C57BL/6 mouse model with chronic MC-LR exposure via drinking water to explore the potential mechanism underlying MC-LR-induced gastric toxicity.

## 2. Results

### 2.1. Characteristics

**Figure 1:**
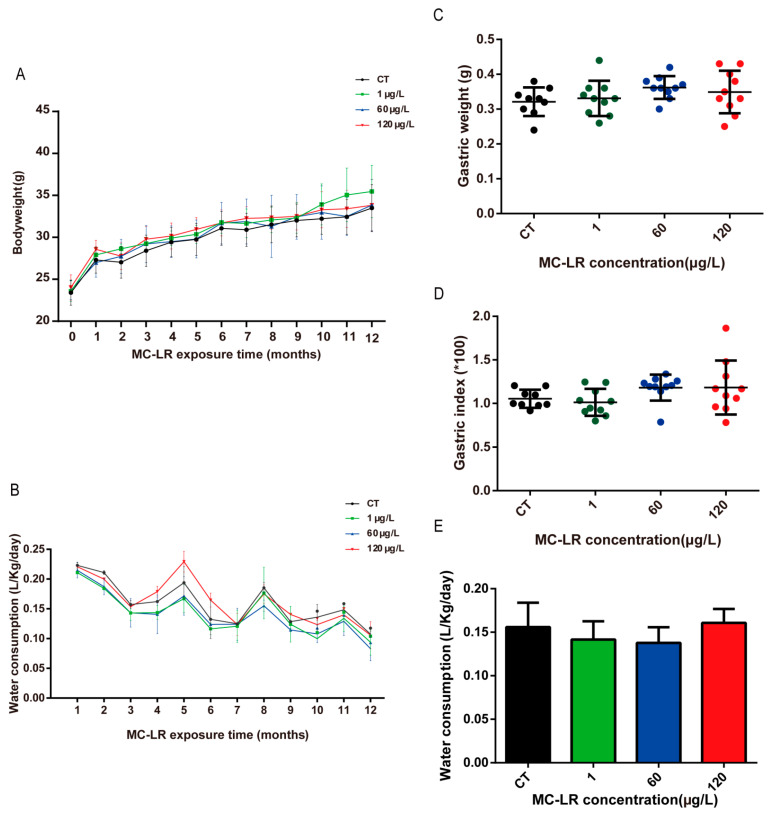
Characteristics. (**A**) Body weight; (**B**) water consumption; (**C**) gastric weight; (**D**) gastric index; (**E**) water consumption of the 12th month. Data are presented as mean ± SD (*n* = 10).

No mortality was observed in the mice during the whole experimental period. During the whole experiment period, the body weight of all mice showed a rising trend. The mice had no significant difference in body weight during the entire experiment (Figure 1A, *p* > 0.05). No difference was observed in water consumption among the MC-LR exposure groups, and the average daily water intake per mouse was about 3 mL, approximately 0.15 L/kg/day (Figure 1B,E, *p* > 0.05). The gastric tissue index of the experimental mice was calculated using the following formula: gastric index (%) = gastric weight/body weight × 100% [26]. After 12 months of MC-LR exposure, no significant difference was found in gastric tissue weight and gastric index among all the groups (Figure 1C,D, *p* > 0.05).

### 2.2. MC-LR Concentration in Gastric Tissue

**Figure 2:**
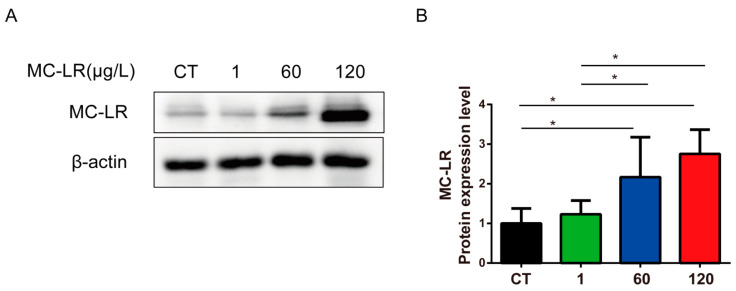
MC-LR concentration in gastric tissue. (**A**) Western blot results, and (**B**) quantitative results of MC-LR through Western blot. Data are presented as mean ± SD (*n* = 3). * indicates significant difference between two groups (*p* < 0.05).

MC-LR expression was detected in the gastric tissue of mice (Figure 2). The obtained data showed that the expression of MC-LR protein in the gastric tissues of mice exposed to the MC-LR was notably upregulated compared to that in the CT mice (Figure 2, *p* < 0.05), and the concentration of MC-LR in the gastric tissues of mice increased correspondingly with the increase in MC-LR concentrations, especially in the 60 µg/L and 120 µg/L exposure groups. MC-LR expression was markedly increased in the 60 µg/L and 120 µg/L groups. But there was no significant difference found between the 60 µg/L and 120 µg/L groups.

### 2.3. Histopathology in the Stomach

**Figure 3:**
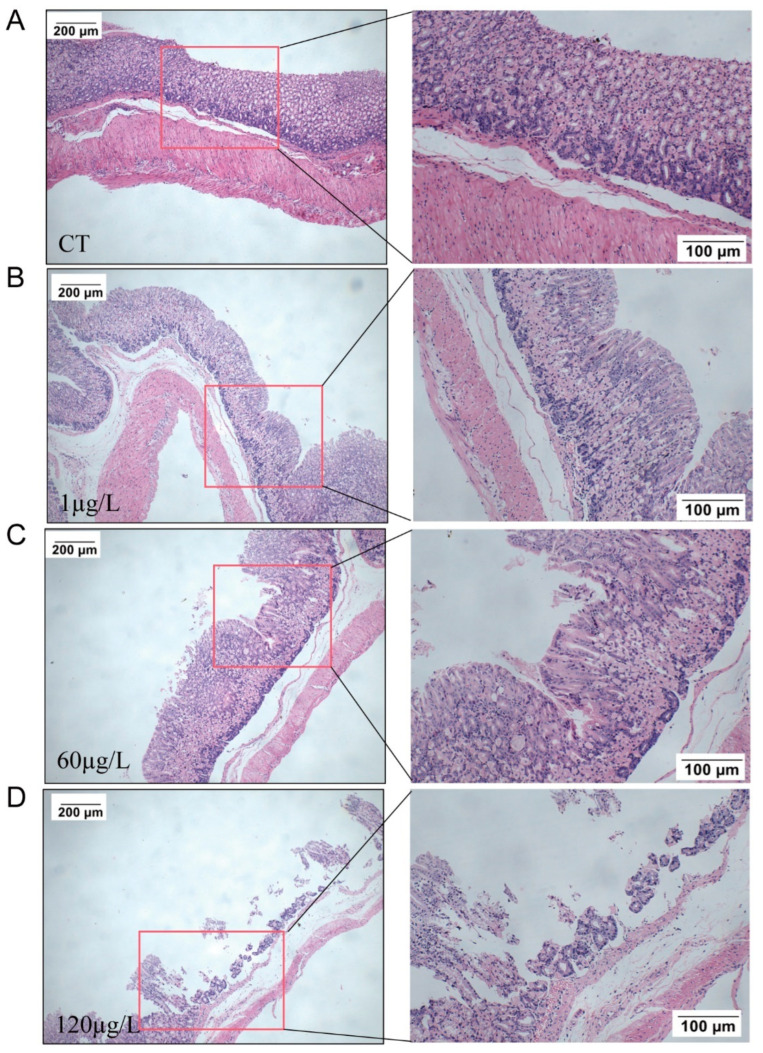
Representative histopathological images of mice gastric tissue: Normal control (CT) groups (**A**); 1 µg/L group (**B**); 60 µg/L group (**C**); 120 µg/L group (**D**).

After 12 months of exposure, histopathological changes were observed in HE-stained pathological sections of gastric tissue. The results showed that with an increase in the dose of MC-LR exposure, the muscular layer of the mucosa in the gastric gland area became thinner, and the severity of shedding of the mucosal layer increased. Ulcer foci appeared in the 60 µg/L MC-LR exposure group, and the mucosal layer fell off in the 120 µg/L MC-LR exposure group (Figure 3), indicating that MC-LR exposure caused gastric tissue barrier disruption.

### 2.4. Tight-Junction-Related Genes and Protein Expression of Gastric Tissue

**Figure 4:**
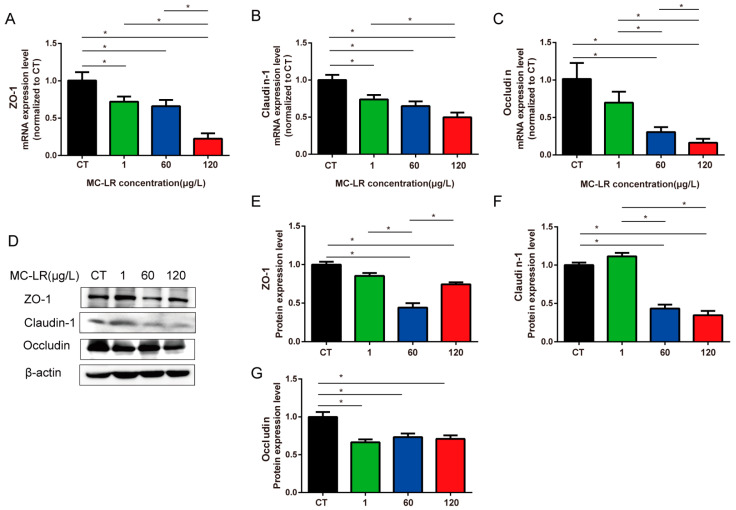
qPCR and Western blot detected tight junction marker factor expression. The mRNA expression of *ZO-1* (**A**), *Claudin-1* (**B**), and *Occludin* (**C**), and representative Western blot results (**D**). The quantitative results of ZO-1, Claudin-1, and Occludin, respectively (**E**–**G**). Data are presented as mean ± SD (*n* = 3). * indicates significant difference between two groups (*p* < 0.05).

There was a notable decrease in the mRNA levels of *ZO-1* and *Claudin-1* in the gastric tissues of MC-LR mice, including the 1, 60, and 120 µg/L groups (Figure 4A,B, *p* < 0.05). In comparison with the CT group, the *Occludin* gene expression was significantly down-regulated in the gastric tissues of the 60 and 120 µg/L MC-LR-exposed groups. Moreover, there was a significant difference between the 60 µg/L and 120 µg/L groups (Figure 4C, *p* < 0.05). ZO-1, Claudin-1, and Occludin protein expressions were significantly down-regulated in the gastric tissues of the 60 and 120 µg/L MC-LR-exposed mice in comparison with the CT mice (Figure 4E–G, *p* < 0.05). In addition, compared with the 120 µg/L group, ZO-1 protein expression was notably decreased in the 60 µg/L group (Figure 4E, *p* < 0.05). Claudin-1 and Occludin protein expression was markedly decreased in the 60 µg/L and 120 µg/L groups. But there was no significant difference between the two groups (Figure 4F,G, *p* < 0.05). The above results suggested that MC-LR exposure might disrupt tight junctions by down-regulating the mRNA and protein expression of ZO-1, Claudin-1, and Occludin, contributing to gastric barrier damage.

### 2.5. Inflammatory Cytokine mRNA Expression in Gastric Tissue of Mice

**Figure 5:**
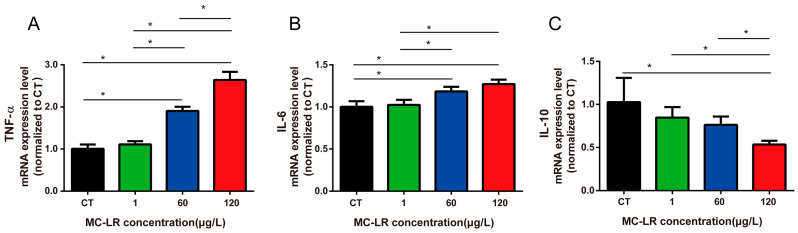
MC-LR affected mRNA expression of *TNF-ɑ* (**A**), *IL-6* (**B**), and *IL-10* (**C**) in mouse gastric tissue. Data are presented as the mean ± SD (*n* = 3). * indicates significant difference between two groups (*p* < 0.05).

Gastric barrier damage was closely associated with inflammation, so we further assessed the inflammation-related factors in the mice’s stomachs. As shown in Figure 5, pro-inflammatory cytokines were significantly increased, including *TNF-α* and *IL-6* (Figure 5A,B, *p* < 0.05), while *IL-10*, the anti-inflammatory cytokines, was considerably decreased in the 120 µg/L MC-LR group (Figure 5C, *p* < 0.05), indicating that MC-LR-induced gastric inflammation was associated with the upregulation of *TNF-α* and *IL-6* mRNA expression and the downregulation of *IL-10* mRNA level.

### 2.6. MAPK Signaling Pathway and Mitophagy-Related Proteins Expression

**Figure 6:**
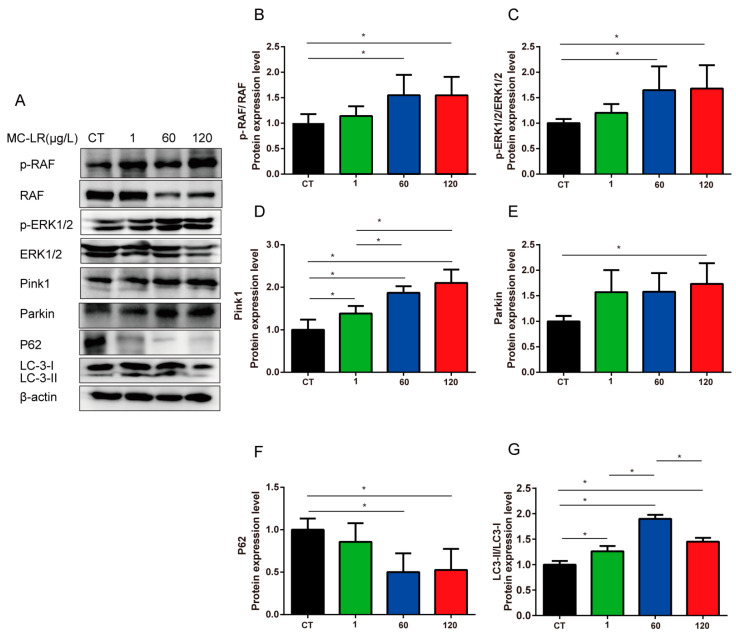
MC-LR promoted MAPK signaling pathway and mitophagy in mice gastric tissue. (**A**) Protein levels of RAF, p-RAF, ERK1/2, p-ERK1/2, Pink1, Parkin, P62, LC3, and β-actin; (**B**) p-RAF protein; (**C**) p-ERK1/2 protein; (**D**) Pink1 protein; (**E**) Parkin protein; (**F**) P62 protein; and (**G**) LC3-II protein. Data are presented as mean ± SD (*n* = 3). * indicates significant difference between two groups (*p* < 0.05).

We determined the protein levels of the MAPK signaling pathway, including p-RAF, RAF, p-ERK1/2, and ERK1/2 proteins. Compared with the CT group, the relative protein expression of p-RAF/RAF and p-ERK1/2/ERK1/2 proteins increased significantly in the 60 and 120 µg/L MC-LR exposure groups (Figure 6A–C, *p* < 0.05). Previous studies have shown that the MAPK signaling pathway induced mitophagy [43,44]. Thus, we determined the expression level of mitophagy-related proteins, including Pink1, Parkin, P62, and LC3-II.

The obtained results showed that the protein expression levels of lipid-bound LC3-II in the 60 µg/L MC-LR group was significantly increased in comparison with the CT group. (Figure 6A,G, *p* < 0.05), which was a common autophagy marker protein with levels proportional to the number of autophagosomes. In addition, the expressions of protein Pink1 and Parkin were significantly increased in the 120 µg/L MC-LR exposure group, and the expression of Pink1 in the gastric tissues of mice increased correspondingly with the MC-LR concentrations, especially in the 60 µg/L and 120 µg/L groups (Figure 6D,E, *p* < 0.05). Conversely, the level of p62, a selective autophagy substrate that dissipates under autophagy promotion, was decreased after exposure to MC-LR, suggesting activation of mitophagy (Figure 6F, *p* < 0.05). Taken together, our results indicated that MC-LR exposure might induce gastric toxicity by promoting the MAPK pathways.

## 3. Discussion

The outbreak of cyanobacteria blooms in freshwater bodies has caused serious environmental problems, and the MC-LR produced by them is extremely harmful to human health [1,2]. Recently, a study reported that the serum MC level of fishermen living near Chaohu Lake was up to 0.39 μg/L [47]. Moreover, the content of MCs in the Chaohu Lake water body was 26.7 μg/L, which is far above the WHO guideline [48]. Drinking and touching contaminated water, physical contact, etc., are the major routes of MC-LR exposure [11,49]. And drinking MC-LR-contaminated water is the most common exposure route [50,51]. Studies have focused on the enterotoxicity and hepatotoxicity of chronic MC-LR exposure. However, studies on gastric toxicity induced by MC-LR are limited. Vidal et al. [29] reported that people develop gastrointestinal symptoms after a few hours of exposure to cyanobacteria. However, the molecular mechanisms by which MC-LR exposure adversely affects the stomach at long-term environmental levels have not been elucidated. Here, we established a mice model of chronic environmental MC-LR exposure, induced gastric injury for the first time, and investigated the underlying toxicological mechanisms.

Earlier studies of MC-LR on gastric toxicity investigated short-term acute exposure in vitro, which was significantly far from the exposure in nature [23,26,27]. In this study, the exposure mode (via drinking water) and the exposure concentration of MC-LR were consistent with the environmental MC-LR exposure level. Studies reported that MC-LR could be detected in gastric tissue after acute MC-LR exposure [26], which was consistent with the present results, and exhibited a dose-dependent trend. In addition, we observed that the mucosal muscle layer in the glandular gastric area became thinner and the mucosal layer fell off when the oral MC-LR dose increased. Similar to our present study, it was reported that MC-LR could induce marked invagination and fold disappearance in the epithelial surface of acutely low-dose-exposed medaka embryos [27]. The mechanical barrier of the gastric mucosa is primarily made up of tightly linked epithelial cells. The formation of tight junctions involves a vast number of tight junction proteins, of which ZO-1, Claudin-1, and Occludin are the primary components [36]. ZO-1 is a key component of TJs that anchors Occludin and Claudin to the actin cytoskeleton [52]. Claudins affect the formation of TJs and the properties of the epithelial barrier [53]. Occludin is vital in the tight junction barrier function of several types of epithelial cells and can be phosphorylated on serine and threonine residues [54,55]. Increased gastrointestinal permeability due to tight junction complex disturbances is related to several diseases, including gastritis and gastric cancer [56]. Our results showed that long-term MC-LR exposure resulted in a significantly reduced expression of mRNA at the transcriptional level of *ZO-1*, *Claudin-1*, and *Occludin*. These results indicated that MC-LR was absorbed in gastric tissue and disrupted the gastric barrier structure, leading to severe gastric toxicity.

Previous studies have shown that tight junctions and inflammation regulate each other. Yokouchi et al. [57] found that tight junction dysfunction can be induced through inflammation in the skin, leading to dysfunction of the barrier. Here, we found that the gastric tissue barriers of 60 and 120 μg/L mice were severely damaged, and the mRNA levels of tightly linked key molecules including *ZO-1*, *Claudin-1*, and *Occludin* were absolutely down-regulated, while *IL-6* and *TNF-ɑ* mRNA levels were up-regulated markedly in the gastric tissues, which were similar to our previous results about the inflammation responses in the colorectum [19]. Notably, our present results showed that MC-LR caused damage to tight junctions and inflammation in the stomach of mice, which has not been reported in previous studies.

MC-LR exerts toxicity primarily by inhibiting serine/threonine-specific protein phosphatase 1 (PP1) and 2A (PP2A) [31,32], mainly regulating protein dephosphorylation [58]. It is responsible for MC’s alteration of cellular metabolism through irreversible inhibition of PP1 and PP2A and leads to symptoms of MC toxicity such as gastroenteritis, irritation, and liver disease [29,59,60]. Irreversible PP1 and PP2A disrupt the protein phosphorylation/dephosphorylation dynamic equilibrium, activating ERK1/2 of the MAPK signaling pathway [41], and leading to damage to the digestive system. MAPKs/ERKs are a class of serine/threonine kinases involved in various cellular and physiological functions [61,62]. As the initial protein kinase in the MAPK signaling pathway, RAF phosphorylates MEK1/2 and further phosphorylates to activate ERK1/2, which then activates transcription factors, leading to the activation of transcription factors [63,64]. A previous study has indicated that the MAPK/ERK1/2 pathway regulates the formation and maintenance of the blood–epididymal barrier in mice [65]. Chen et al. [66] found that MC-LR treatment inhibited the expression of miR-98-5p and miR-758 in Sertoli cells, promoting the protein expression of MAPK11. MAPK11 signaling increased the protein levels of ATF2, thus activating the transcription of TNF-α and thus inducing inflammation. Zhou et al. [67] demonstrated that MC-LR induced intestinal barrier dysfunction by inhibiting PP2A to activate the PI3K/AKT and MAPK signaling pathways, disrupting tight junctions between intestinal epithelial cells, which was consistent with our present study and showed that MC-LR increased key proteins in the MAPK signaling pathway phosphorylation. Therefore, we speculate that the MAPK signaling pathway plays an important role in MC-LR-induced tight junction damage. Further studies are needed to confirm how MC-LR affects TJs in the stomach of mice via the MAPK signaling pathway.

Studies have linked the MAPK signaling pathway to mitophagy [43,44]. The PTEN-induced putative kinase 1(PINK1)/E3 ubiquitin ligase Parkin (PARK2)-mediated pathway weighs more among the two pathways in mitochondrial autophagy, including p62-LC3 and autophagosome formation, and selective proteasomal/lysosomal degradation [68,69,70,71]. Cong et al. [72] revealed that tight junction dysfunction was related to mitophagy, showing a strong negative correlation between the expression of TJ-related proteins (ZO-1, Claudin-1, and Occludin) and the expression of mitophagy-related proteins (Parkin, PINK1, and LC3II/I). In this study, we found that the transcriptional levels of ZO-1, Claudin-1, and Occludin markedly decreased and the protein levels of Parkin, Pink1, and LC3II/I were significantly increased. Therefore, we hypothesized that MC-LR-induced gastric toxicity in the gastric tissues of MC-LR-exposed mice might be related to mitophagy and tight junction dysfunction.

## 4. Conclusions

We first demonstrated that long-term chronic low-dose MC-LR exposure exhibited toxic effects on gastric tissues, manifested by inflammation and gastric barrier disruption. MC-LR absorbed in gastric epithelial cells via OATPs; activated the MAPK signaling pathway via PP1 and PP2A inhibition; significantly decreased the expression of TJ-related genes *ZO-1*, *Claudin-1*, and *Occludin;* but increased the expression of inflammatory genes in the gastric tissue of MC-LR mice, thus inducing dysfunction of the intestinal barrier and inflammation. In addition, tight junction dysfunction might be associated with mitophagy. Our study provided new evidence for the gastric toxic effects and molecular mechanisms caused by MC-LR, which might be useful for the prevention and treatment of gastric toxicity caused by MC-LR. Studies are required for elucidating the exact underlying mechanism.

## 5. Methods and Materials

### 5.1. Animals and Treatments

Approval for all animal studies was obtained from the Animal Care and Use Committee of the Central South University (permit no. XYGW-2018-41). A total of 40 healthy male C57BL/6 mice at 8 weeks of age, weighing 20–22 g, were purchased from Hunan SJA Laboratory Animal Co., Ltd. (Hunan, China). Animals were kept in standard environmental conditions with a 12 h light/dark cycle at the Experimental Animal Center of Central South University. After one week of adaptive feeding, mice were divided into 4 groups randomly and exposed to MC-LR at concentrations of 0, 1, 60, and 120 μg/L MC-LR via drinking water for 12 months, with 10 mice in each group, consistent with our previously published study [19]. Body weight was measured weekly. After 12 months, the mice were fasted for 12 h, and then anesthetized and euthanized. Blood and gastric tissue samples were collected and stored at −80 °C for subsequent detection.

### 5.2. Histology Staining

Gastric tissue hematoxylin and eosin (HE) staining was conducted according to Yang et al. [19].

### 5.3. qRT-PCR

Trizol Reagent (Invitrogen) was used to isolate the total RNA of gastric tissues. The RNA concentration and A260/280 ratio were measured using an Ultra-micro ultraviolet-visible spectrophotometer (MIULAB, Hangzhou, China), and the RNA was reverse-transcribed to cDNA using a reverse transcription kit. Primers were designed in Primer Premier 5.0 software, and the designed primers were validated at NCBI and handed over to Shanghai Biotech, Shanghai, China, for synthesis. Table 1 shows all primer sequences used for the qPCR. And the reaction conditions were according to Yang et al. [19]. Transcription fold changes of target genes were calculated using the 2^−ΔΔCt^ comparison method.

### 5.4. Western Blot

The gastric tissue was lysed in RIPA (Beyotime, Shanghai, China), a protease inhibitor (Cwbiotech, Beijing, China) and phosphatase inhibitor (Cwbiotech, Beijing, China), and enzyme-free grinding beads were added. Protein concentration was detected using the BCA kit (Beyotime, Shanghai, China). Protein samples were separated and transferred to PVDF membranes (Merck Millipore Ltd., Burlington, MA, USA). In addition, 5% skimmed milk powder (Cell Signaling Technology Company, Danvers, MA, USA) and 5% BSA (Biofroxx, Guangzhou, China) were blocked for 2 h. Primary antibodies (Table 2) were incubated overnight at 4 °C in a refrigerator and washed 3 times with tris-buffered saline-tween 20 (TBST). Primary antibodies were used to determine the expression of the following proteins: MC-LR, total RAF and p-RAF, total ERK1/2 and p-ERK1/2, Pink1, Parkin, P62, LC-3, and β-actin. A chemiluminescence system (Bio-Rad, Hercules, CA, USA) was used to determine the protein bands, and the intensity was measured using Image J software (V 1.8.0).

### 5.5. Statistical Analysis

Data are provided as mean ± standard deviation (SD). One-way ANOVA followed by an LSD post hoc test was conducted in SPSS 26.0 software (SPSS Inc., Chicago, IL, USA) to explore differences among groups. *p* < 0.05 was considered statistically significant.

## Figures and Tables

**Table 1:** The primer sequence required for the experiment.

Primer Name	Forward Primer	Reverse Primer
β-actin	TCAAGATCATTGCTCCTCCTGAG	ACATCTGCTGGAAGGTGGACA
TNF-α	GTGCCTATGTCTCAGCCTCT	AGGCTTGTCACTCGAATTTTGA
IL-6	CCACGGCCTTCCCTACTTC	TTGGGAGTGGTATCCTCTGTGA
IL-10	ATAACTGCACCCACTTCCCA	GGGCATCACTTCTACCAGGT
ZO-1	GCGATTCAGCAGCAACAGAACC	AGGACCGTGTAATGGCAGACTC
Occludin	GCGGCTATGGAGGCTATGGCTA	AGGAAGCGATGAAGCAGAAGGC
Claudin-1	GGACAACATCGTGACCGCTCAG	TCCAGGCACCTCATGCACTTCA

**Table 2:** Antibodies.

Antibody Name	Source	Dilution Ratios
MC-LR	Alexis Corporation (Lausen, Switzerland)	1:3000
RAF	Proteintech, Wuhan, China	1:1000
p-RAF	Cell Signaling Technology Company, USA	1:1000
ERK	Proteintech, Wuhan, China	1:1000
p-ERK	Cell Signaling Technology Company, USA	1:1000
Pink-1	Proteintech, Wuhan, China	1:1000
Parkin	Abcam, The United Kingdom	1:1000
P62	Proteintech, Wuhan, China	1:1000
LC-3	Proteintech, Wuhan, China	1:500
β-actin	Proteintech, Wuhan, China	1:1000

## Data Availability

The data presented in this study are available in this article.
